# Targeting cancer stem cell propagation with palbociclib, a CDK4/6 inhibitor: Telomerase drives tumor cell heterogeneity

**DOI:** 10.18632/oncotarget.14196

**Published:** 2016-12-25

**Authors:** Gloria Bonuccelli, Maria Peiris-Pages, Bela Ozsvari, Ubaldo E. Martinez-Outschoorn, Federica Sotgia, Michael P. Lisanti

**Affiliations:** ^1^ Paterson Building, University of Manchester, Manchester M20 4BX, United Kingdom; ^2^ The Sidney Kimmel Cancer Center, Thomas Jefferson University, Philadelphia, Pennsylvania 19107, USA; ^3^ Translational Medicine, School of Environment and Life Sciences, Biomedical Research Centre (BRC), University of Salford, Greater Manchester, M5 4WT, United Kingdom

**Keywords:** cancer stem-like cells (CSCs), telomerase, doxycycline, palbociclib, mitochondria, tumor dormancy

## Abstract

In this report, we systematically examined the role of telomerase activity in lung and ovarian cancer stem cell (CSC) propagation. For this purpose, we indirectly gauged telomerase activity, by linking the hTERT-promoter to eGFP. Using lung (A549) and ovarian (SKOV3) cancer cells, transduced with the hTERT-GFP reporter, we then employed GFP-expression levels to fractionate these cell lines into GFP-high and GFP-low populations. We functionally compared the phenotype of these GFP-high and GFP-low populations. More specifically, we now show that the cancer cells with higher telomerase activity (GFP-high) are more energetically activated, with increased mitochondrial mass and function, as well as increased glycolytic activity. This was further validated and confirmed by unbiased proteomics analysis. Cells with high telomerase activity also showed an increased capacity for stem cell activity (as measured using the 3D-spheroid assay) and cell migration (as measured using a Boyden chamber approach). These enhanced biological phenotypes were effectively inhibited by classical modulators of energy metabolism, which target either i) mitochondrial metabolism (i.e., oligomycin) or ii) glycolysis (i.e., 2-deoxy-glucose), or iii) by using the FDA-approved antibiotic doxycycline, which inhibits mitochondrial biogenesis. Finally, the level of telomerase activity also determined the ability of hTERT-high cells to proliferate, as assessed by measuring DNA synthesis via EdU incorporation. Consistent with these observations, treatment with an FDA-approved CDK4/6 inhibitor (PD-0332991/palbociclib) specifically blocked the propagation of both lung and ovarian CSCs. Virtually identical results were obtained with breast CSCs, which were also highly sensitive to palbociclib at concentrations in the nanomolar range. In summary, CSCs with high telomerase activity are among the most energetically activated, migratory and proliferative cell sub-populations. These observations may provide a mechanistic explanation for tumor metabolic heterogeneity, based on telomerase activity. FDA-approved drugs, such as doxycycline and palbociclib, were both effective at curtailing CSC propagation. Thus, these FDA-approved drugs could be used to target telomerase-high proliferative CSCs, in multiple cancer types. Finally, our experiments also allowed us to distinguish two different cellular populations of hTERT-high cells, one that was proliferative (i.e., replicative immortality) and the other that was non-proliferative (i.e., quiescent). We speculate that the non-proliferative population of hTERT-high cells that we identified could be mechanistically involved in tumor dormancy.

## INTRODUCTION

Telomerase is a critical enzyme that serves an important functional role in a plethora of biological processes, ranging from organismal development and regeneration to chronological aging [1] and the onset of the malignant phenotype in human cancers [2–4]. Yet, despite years of study, many of the functions of telomerase still remain unknown or poorly defined. Mechanistically, telomerase consists of both RNA-based and proteinaceous components or subunits: telomerase RNA (TERC) and telomerase reverse transcriptase (TERT) [5]. High levels of telomerase expression are observed in normal stem cells [6] and cancer cells [7]. In both these normal and pathological contexts, telomerase expression functionally confers immortalization [8], allowing cells to bypass senescence and to multiply past fifty to seventy divisions [9].

Several research groups have now exploited human telomerase (hTERT) to allow for the enrichment of cancer stem-like cells (CSCs). More specifically, Yu et al., 2013 [10], placed a 1.5-kB fragment of the hTERT promoter upstream of eGFP. Recombinant transduction of this hTERT-eGFP reporter into osteosarcoma cell lines then allowed the purification of a telomerase enriched cell population by flow cytometry. They also directly showed that hTERT-high cells derived from an osteosarcoma cell line were more stem-like and underwent anchorage-independent growth. Furthermore, the hTERT-high osteosarcoma cell population was more invasive, with a greater capacity for drug-resistance and metastatic dissemination. Thus, this innovative approach to CSC enrichment has already shown great promise [11], but needs to be further validated and extended to the characterization of various epithelial cancer types, such as non-small cell lung cancer and ovarian carcinomas, among others.

In this report, we applied this general approach to two independent cell lines, namely A549 and SKOV3 cells, which were originally derived from human cancer patients suffering from lung and ovarian cancer, respectively. Then, we focused on the functional and molecular characterization of the hTERT-high cell population isolated by flow cytometry. More specifically, we employed unbiased proteomics analysis [12, 13] and validation via functional metabolic studies, which allowed us to identify new therapeutic strategies for the eradication of the hTERT-high cell population.

## RESULTS

Here, we used an established approach based on telomerase activity, to begin to dissect the role of hTERT in tumor cell heterogeneity. Briefly, A549 (lung) and SKOV3 (ovarian) cancer cell lines were stably-transduced with a sensitive eGFP reporter system for the fluorescent detection of high telomerase transcriptional activity. This allowed us to isolate the hTERT-high cell population by flow cytometry. The phenotypic behavior of GFP(+) and GFP(-) populations was then compared quantitatively. In this analysis, the GFP(+) cell population represents the hTERT-high cell population, while the GFP(-) cells serve as a critical internal control for phenotypic comparison.

### hTERT-high CSCs show elevated levels of ALDH activity and higher mitochondrial mass

**Figure 1:**
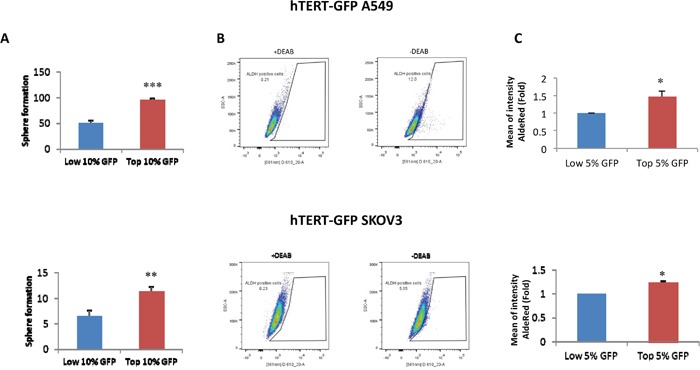
Validation of the cellular models: hTERT-high cells show an increased capacity for 3D-sphere formation and are enriched in ALDH activity **A**. 3D-sphere formation. A549 and SKOV3 cells stably-transduced with the hTERT-eGFP reporter were subjected to FACS sorting to isolate the GFP(+) and GFP(-) cell populations. GFP(+) cells were operationally defined as those with the top 10% eGFP intensity, while GFP(-) cells were defined as those with the lowest 10% eGFP intensity, unless stated otherwise. The GFP-high and GFP-low cell populations were then plated in low-attachment plates for 3D-spheroid assays and analyzed after 5 days of culture. Note that GFP(+) A549 and GFP(+) SKOV3 cells both form 3D-spheroids with a near 2-fold increased efficiency, as compared to their corresponding GFP(-) counterparts. **B** and **C**. ALDH activity profile. A549 and SKOV3 cells stably-transduced with the hTERT-eGFP reporter were subjected to FACS sorting to isolate the GFP(+) and GFP(-) cell populations. GFP(+) cells were operationally defined as those with the top 5% eGFP intensity, while GFP(-) cells were defined as those with the lowest 5% eGFP. Then, they were counter-stained with AldeRed to identify the ALDH(+) cell population. Representative tracings are shown in panel B, in the presence or absence of the ALDH inhibitor (DEAB). Panel C shows that the GFP(+) cell populations were enriched in ALDH activity relative to the GFP(-) cell population. Therefore, higher telomerase activity correlates with increased 3D-spheroid formation and increased ALDH activity. ALDH intensity is expressed as mean fluorescence intensity of 3 experiments. *N*=3 independent experiments, with 2 technical replicates per experiment. Bar graphs are shown as the mean ± SEM; *t*-test, two-tailed test *p < 0.05, **p < 0.005, ***p < 0.0001.

Figure 1A shows that hTERT-high cells form 3D-spheroids (>50-μm) with a greater efficiency, up to 2-fold higher, as observed by comparing the GFP(+) and GFP(-) cell populations. Virtually identical results were obtained with both A549 and SKOV3 cell lines, indicating that this is a conserved property of hTERT-high CSCs. Similarly, hTERT-high cells were significantly enriched in ALDH-activity, a well-established marker of CSCs (Figure 1B, 1C).

**Figure 2:**
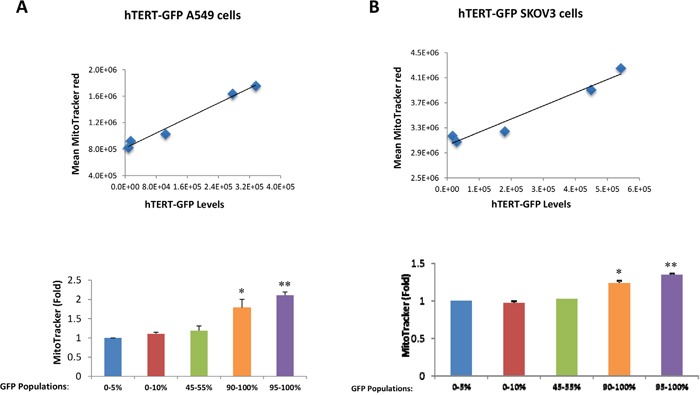
hTERT-high cells show a significant increase in mitochondrial mass A549 and SKOV3 cells stably-transduced with the hTERT-eGFP reporter were subjected to flow cytometry to identify the GFP(+) and GFP(-) cell populations. In addition, the cells were also counter-stained with MitoTracker Deep Red to correlate their mitochondrial mass with GFP intensity. Five different cell populations with different GFP intensities were considered in this analysis. Note that **panel A** shows that mitochondrial mass increases with increasing GFP levels in hTERT-GFP A549 cells. **Panel B** illustrates that similar results were observed with hTERT-GFP SKOV3 cells. Therefore, higher telomerase activity correlates with increased mitochondrial mass in both cell lines. *N*=3 independent experiments, with 2 technical replicates for each experiment. Bar graphs are shown as the mean ± SEM; *t*-test, two-tailed test *p < 0.05, **p < 0.001.

**Figure 3:**
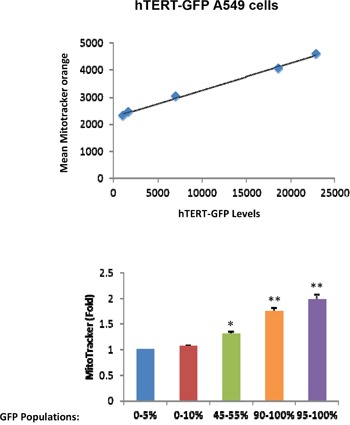
hTERT-high cells show a significant increase in mitochondrial membrane potential/activity Cell populations were analyzed as outlined in Figure 2, except that MitoTracker Orange was used as a marker of mitochondrial membrane potential. Note that in A549 cells, mitochondrial membrane potential increases with increasing GFP levels. Therefore, higher telomerase activity correlates with increased mitochondrial membrane potential in A549 cells. *N*=3 independent experiments, 2 technical replicates per experiment. Bar graphs are shown as the mean ± SEM; *t*-test, two-tailed test *p < 0.05, **p < 0.001.

Because hTERT is mechanistically linked to increased mitochondrial biogenesis [11], we next examined the status of mitochondrial mass in hTERT-high cells. For this purpose, we directly correlated the eGFP fluorescence signal (representing hTERT activity) with the intensity of MitoTracker Deep Red (a marker of mitochondrial mass). Importantly, Figure 2 demonstrates that GFP(+) hTERT-high cells have a higher mitochondrial mass, as predicted [11]. Virtually identical results were also obtained with another mitochondrial probe, namely MitoTracker Orange, which provides a measure of mitochondrial membrane potential (Figure 3).

**Figure 4:**
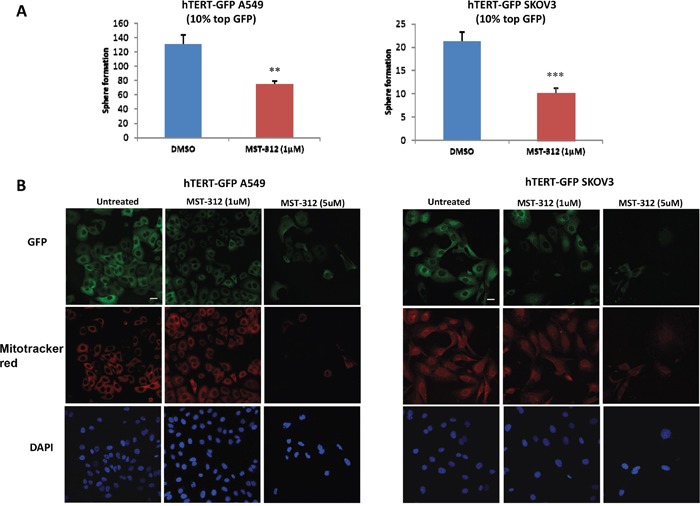
Inhibition of telomerase activity effectively reduces 3D-spheroid formation, GFP intensity and mitochondrial mass **A**. 3D-sphere formation A549 and SKOV3 cells stably-transduced with the hTERT-eGFP reporter were subjected to FACS sorting to isolate the GFP(+) cell population. Then, the phenotype of the GFP(+) population was analyzed in the absence or presence of the telomerase inhibitor. Note that treatment with the telomerase-inhibitor MST-312 (1 μM) decreased 3D-sphere formation in GFP(+) A549 cells (1.75 fold, left panel) and in GFP(+) SKOV3 cells (2 fold, right panel). Bar graphs are shown as the mean ± SEM; t-test, two-tailed test **p < 0.005, ***p < 0.0001. **B**. Immuno-fluorescence analysis. Treatment of adherent cells with the telomerase-inhibitor MST-312 (1 μM and 5 μM) decreases GFP and MitoTracker Deep Red staining, in a dose-dependent fashion. Representative immunofluorescence images show that 1 μM MST-312 causes a slight decrease of the GFP and MitoTracker signal in both cell lines. A more dramatic effect is observed after treatment with 5 μM MST-312 in both hTERT-GFP A549 cells and hTERT-GFP SKOV3 cells. Original magnification, 40x. Scale bar 20 μm. Therefore, 3D-sphere formation, GFP expression and mitochondrial mass are strictly dependent on telomerase activity, as predicted.

As a further test of the specificity of this experimental system, we next employed a well-established telomerase inhibitor (MST-312; 1 μM) [10]. Figure 4A shows that treatment with MST-312 significantly reduces the ability of hTERT-high cells to form 3D-spheroids (a measure of anchorage-independent growth capacity). Consistent with this finding MST-312 treatment also reduces the expression of eGFP and mitochondrial mass in the hTERT-high cell population, as seen by fluorescence microscopy (Figure 4B). Thus, eGFP intensity does indeed reflect telomerase activity, and is functionally connected to mitochondrial mass [11].

### hTERT-high CSCs are more metabolically active and their propagation and migration are inhibited by targeting mitochondrial function

**Figure 5:**
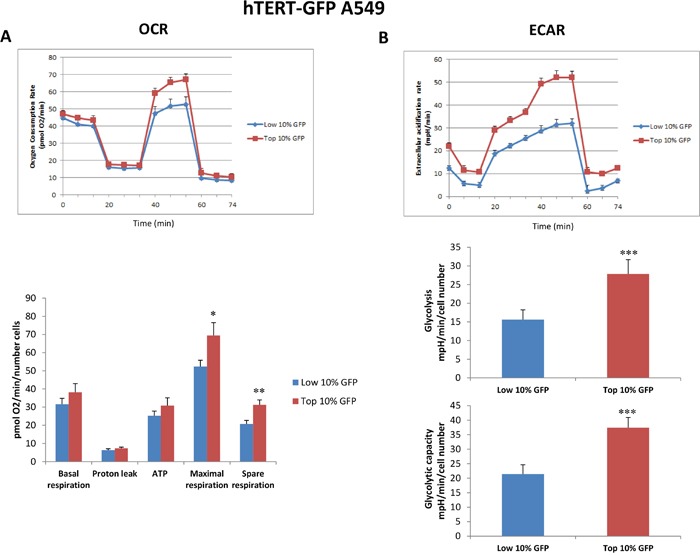
Mitochondrial respiration and glycolysis are both significantly enhanced in hTERT-high A549 cells A549 cells stably-transduced with the hTERT-eGFP reporter were subjected to FACS sorting to isolate the GFP(+) and GFP(-) cell populations. Then, the metabolic profiles of these 2 cell populations was then analyzed quantitatively. Briefly, hTERT-GFP A549 sup-populations were isolated by sorting based on GFP levels, and grown as monolayers for 20 hours, prior to metabolic analysis using the Seahorse XFe96 analyzer. **A**. Oxygen consumption. A representative tracing of an OCR experiment is shown in the top panel. The bar graphs in the lower panel show that maximal respiration is increased in GFP(+) cells as compared to GFP(-) cells by 1.32 fold. Also, the spare respiratory capacity is significantly higher in GFP(+) cells by 1.5 fold. Data are presented in the bar graphs as a combination of *N*=3 independent experiments. Bar graphs are shown as the mean ± SEM, *t*-test, two-tailed test. **B**. Glycolysis rate. A representative tracing of an ECAR experiment is shown in the top panel. The bar graph in the lower panel shows that the glycolysis (measured after addition of glucose) is increased in GFP(+) cells as compared to GFP(-) cells by 1.78 fold, and the glycolytic capacity (measured after oligomycin addition) is increased by 1.74 fold. Data are presented in the bar graphs as a combination of *N*=3 independent experiments. Bar graphs are shown as the mean ± SEM; *t*-test, two-tailed test *p < 0.05, **p < 0.005, ***p < 0.0001.

**Figure 6:**
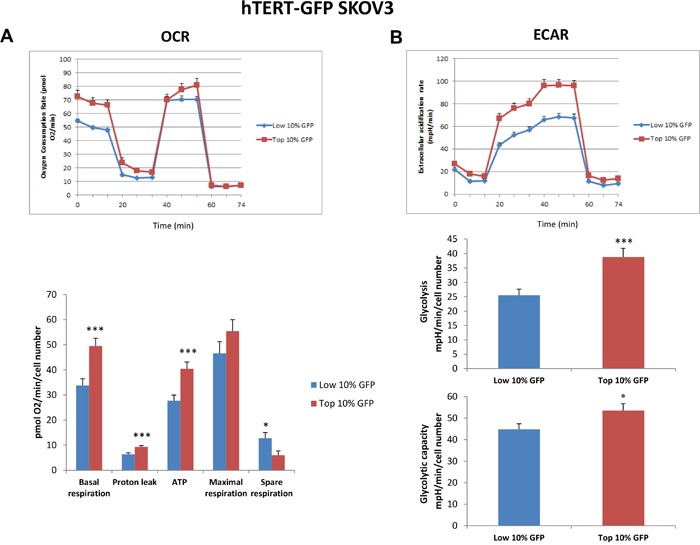
Mitochondrial respiration and glycolysis are both significantly enhanced in hTERT-high SKOV3 cells SKOV3 cells stably-transduced with the hTERT-eGFP reporter were subjected to FACS sorting to isolate the GFP(+) and GFP(-) cell populations. Then, the metabolic profiles of these 2 cell populations was then analyzed quantitatively. Briefly, hTERT-GFP SKOV3 sup-populations were isolated by sorting based on GFP levels, and grown as monolayers for 20 hours, prior to metabolic analysis using the Seahorse XFe96 analyzer. **A**. Oxygen consumption. A representative tracing of an OCR experiment is shown in the top panel. The bar graphs in the lower panel shows that basal respiration, proton leak and ATP production are all increased in GFP(+) cells as compared to GFP(-) cells. Data are presented in the bar graphs as a combination of *N*=3 independent experiments. Bar graphs are shown as the mean ± SEM; *t*-test, two-tailed test. **B**. Glycolysis rate. A representative tracing of an ECAR experiment is shown in the top panel. The bar graphs in the lower panel show glycolysis (measured after addition of glucose) is increased in GFP(+) cells as compared to GFP(-) cells by 1.5 fold, and the glycolytic capacity (measured after oligomycin addition) is also increased by 1.2 fold. Data are presented in the bar graphs as a combination of *N*=5 independent experiments. Bar graphs are shown as the mean ± SEM, *t*-test, two-tailed test. *p < 0.05, ***p < 0.0001.

To better dissect the role of hTERT activity in tumor cell metabolism, we next employed the Seahorse XFe96 bioenergetic analyzer, to directly measure metabolic flux. For this purpose, we systematically compared the metabolic properties of the GFP(+) and GFP(-) cell populations. Figures 5 and 6 illustrate that the GFP(+) population is clearly more metabolically active than the GFP(-) cells, with significant increases in both oxygen consumption and glycolytic rates. This is consistent with the increase in mitochondrial mass that we observed experimentally using the MitoTracker probes. Moreover, unbiased proteomics analysis of the GFP(+) and the GFP(-) cell populations revealed that hTERT-high cells show the up-regulation of key mitochondrial proteins and glycolytic enzymes. These results are summarized in Tables 1 and 2. Importantly, note that the expression of the MT-CO2 protein was significantly increased (by ∼4 to 6-fold) in GFP(+) cell populations. Since MT-CO2 is specifically encoded by mitochondrial DNA, this finding is indicative of an increased capacity for driving new mitochondrial biogenesis. Interestingly, a comparison of Tables 1 and 2 indicates that the following eight metabolism-related proteins were commonly upregulated in hTERT-high cells, derived from both the A549 and SKOV3 cell populations (AK2, ATP5B, HSPA9, HSPD1, LDHB, MT-CO2, PRKDC, and PYGB) (See Supplemental Table 1). These metabolism-related gene products are likely to be specific targets of hTERT in CSCs, and are mainly related to mitochondrial biogenesis.

**Table 1:** Proteomics: Key Molecules Up-regulated in hTERT-GFP(+) A549 Lung Cancer Cells Isolated by FACS

Symbol	Description	Fold-Upregulation(GPF(+)/GFP(-))
**Mitochondrial-related proteins**		
ALDH2	Aldehyde dehydrogenase, mitochondrial	Infinity
AK2	Adenylate kinase 2, mitochondrial	27.20
ATP5B	ATP synthase subunit beta	9.48
IMMT	Mitochondrial inner membrane protein	9.20
MRPL49	Mitochondrial ribosomal protein L49	7.12
PRKDC	DNA-dependent protein kinase catalytic subunit	6.26
HSPA9	Heat shock 70 kDa protein 9	6.00
MT-CO2	Cytochrome c oxidase subunit 2, mt-DNA encoded	3.99
NDUFS2	NADH-ubiquinone oxidoreductase 49 kDa subunit, mitochondrial	3.76
ETFA	Electron transfer flavoprotein subunit alpha, mitochondrial	2.83
SLC25A10	Mitochondrial dicarboxylate carrier	2.39
ATP5O	ATP synthase subunit O, mitochondrial	2.36
VDAC1	Voltage-dependent anion-selective channel protein 1	2.27
SLC25A5	Mitochondrial Carrier; Adenine Nucleotide Translocator, Member 5	1.99
HSPD1	60 kDa heat shock protein, mitochondrial	1.98
**Glycolysis and PPP**		
LDHB	L-lactate dehydrogenase B	50.93
G6PD	Glucose-6-phosphate 1-dehydrogenase	17.85
TPI1	Triosephosphate isomerase 1	17.41
PGK1	Phosphoglycerate kinase 1	9.49
ALDOA	Fructose-bisphosphate aldolase	3.32
PGD	6-phosphogluconate dehydrogenase, decarboxylating	3.21
PKM2	Pyruvate kinase	1.93
**Glycogen-related**		
PYGB	Glycogen phosphorylase, brain form	2.23

**Proteomic analysis of hTERT-GFP A549 cells**. Data are derived from the analysis of FACS sorted cell populations. The fold-increase of certain key molecules in GFP(+) cells is shown, relative to GFP(-) cells. Note that several protein targets related to mitochondria, glycolysis, and glycogenolysis are increased in GFP(+) cells.

**Table 2:** Proteomics: Key Molecules Up-regulated in hTERT-GFP(+) SKOV3 Ovarian Cancer Cells Isolated by FACS

Symbol	Description	Fold-Upregulation(GPF(+)/GFP(-))
**Mitochondrial-related proteins**		
MRPL15	Mitochondrial ribosomal protein L15	11.65
SLC25A10	Mitochondrial dicarboxylate carrier	10.11
DUT	Deoxyuridine 5′-triphosphate nucleotidohydrolase, mitochondrial	9.48
VDAC2	Voltage-dependent anion channel 2	7.23
COX5A	Cytochrome c oxidase subunit 5A, mitochondrial	7.08
MT-CO2	Cytochrome c oxidase subunit 2, mt-DNA encoded	6.08
PRKDC	DNA-dependent protein kinase catalytic subunit	5.90
SHMT2	Serine hydroxymethyltransferase, mitochondrial	5.71
HSPD1	60 kDa heat shock protein, mitochondrial	5.44
FECH	Ferrochelatase, mitochondrial	5.23
ATP5B	ATP synthase subunit beta	4.54
SOD2	Superoxide dismutase 2	4.27
YARS2	Tyrosine--tRNA ligase, mitochondrial	4.18
DLST	Dihydrolipoyllysine-residue succinyltransferase component of 2-oxoglutarate dehydrogenase complex, mitochondrial	4.10
TUFM	Elongation factor Tu, mitochondrial	4.00
ALDH1B1	Aldehyde dehydrogenase X, mitochondrial	3.27
PMPCA	Mitochondrial-processing peptidase subunit alpha	3.25
CS	Citrate synthase	3.25
MTCH2	Mitochondrial carrier homolog 2	3.10
ECI1	Enoyl-CoA delta isomerase 1, mitochondrial	2.41
AK2	Adenylate kinase 2, mitochondrial	2.41
SDHA	Succinate dehydrogenase flavoprotein subunit, mitochondrial	2.39
HSPA9	Stress-70 protein, mitochondrial	2.38
ACO2	Aconitase 2, mitochondrial	1.82
IDH3A	Isocitrate dehydrogenase [NAD] subunit alpha, mitochondrial	1.52
**Glycolysis and PPP**		
LDHAL6B	L-lactate dehydrogenase A-like 6B	8.51
PGK2	Phosphoglycerate kinase 2	6.91
ENO3	Enolase	4.16
PKLR	Pyruvate kinase isozymes R/L	3.71
ENO2	Enolase	3.56
HK1	Hexokinase-1	3.25
LDHB	L-lactate dehydrogenase B	3.08
**Glycogen-related**		
PYGB	Glycogen phosphorylase, brain form	1.63

**Proteomic analysis of hTERT-GFP SKOV3 cells**. Data are derived from the analysis of FACS sorted cell populations. The fold-increase of certain key molecules in GFP(+) cells is shown, relative to GFP(-) cells. Note that several protein targets related to mitochondrial metabolism and glycolysis are increased in GFP(+) cells.

**Figure 7:**
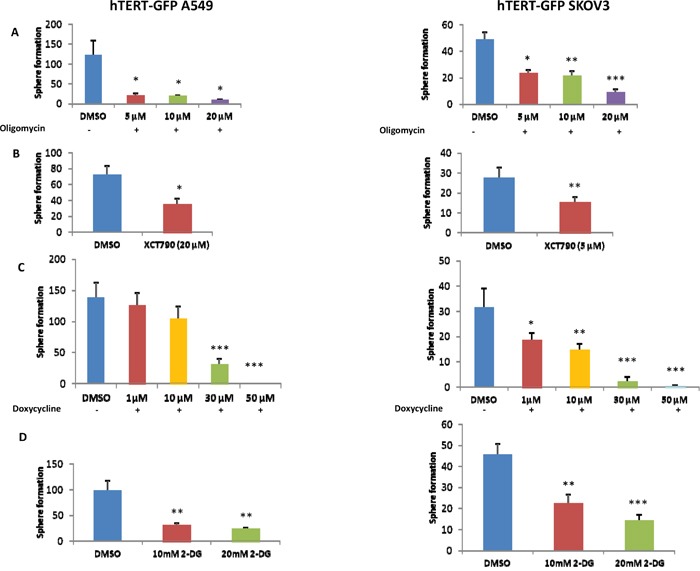
3D-spheroid formation relies both on mitochondrial function and glycolytic metabolism **A-C**. Mitochondrial inhibitors. Mitochondrial function is required for the efficient clonal expansion and anchorage-independent growth of CSCs. Indeed, oligomycin A, an inhibitor of the mitochondrial ATP synthase, inhibits 3D-sphere formation in the GFP(+) cell population isolated from A549 and SKOV3 cells (**panel A**). Complementary results were also obtained with two other well-established mitochondrial inhibitors, namely XCT790 (**panel B**) and doxycycline (**panel C**). XCT790 is a well-established ERRα inverse agonist that effectively blocks the function of PGC1A, the major mitochondrial transcription factor. Doxycycline functions as an inhibitor of mitochondrial biogenesis, by inhibiting mitochondrial protein synthesis at the level of the mito-ribosome. **D**. Inhibition of glycolysis. Glycolytic metabolism is important for 3D-spheroids. Note that treatment with the glycolytic inhibitor 2-deoxy-glucose (2-DG) significantly reduces sphere formation. Bar graphs show the mean ± SEM, *t*-test, two-tailed test. *p < 0.05, **p < 0.005, ***p < 0.001.

To functionally validate the role of oxidative mitochondrial metabolism and glycolysis in the propagation of hTERT-high cells, we used a battery of well-established metabolic inhibitors, to target these essential metabolic pathways. To inhibit mitochondrial function, we employed i) oligomycin (an ATP-synthase inhibitor), ii) XCT790 (an ERRA/PGC1A inhibitor) and iii) doxycycline (an inhibitor of mitochondrial protein synthesis). Similarly, for inhibiting glycolysis, we utilized 2-deoxy-glucose (2-DG). Briefly, 2-DG is a chemically-modified form of glucose in which the 2-hydroxyl group is replaced by hydrogen; as a consequence, it cannot undergo further glycolysis. Figure 7 shows that both inhibitors of glycolysis and mitochondrial metabolism were similarly effective in halting the propagation of hTERT-high cells. This may be related to the fact that glycolysis inhibitors will also ultimately reduce oxidative mitochondrial metabolism, as the final product of glycolysis (pyruvate) is further metabolized by mitochondria.

**Figure 8:**
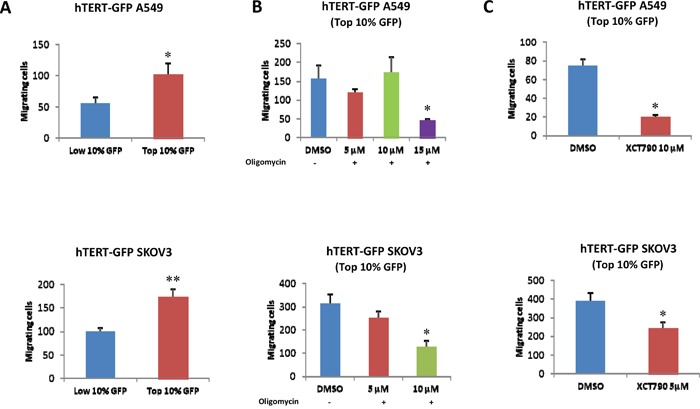
hTERT-high cells show an increased capacity for cell migration, which is strictly dependent on mitochondrial function **A**. Cell migration. A549 and SKOV3 cells stably-transduced with the hTERT-eGFP reporter were subjected to flow cytometry to isolate the GFP(+) and GFP(-) cell populations. Then, the migratory capacity of these two cell populations was assessed using a modified “Boyden Chamber” assay. More specifically, the cells were allowed to migrate across an 8 μm pore uncoated membrane for 12-16 hours. Note that the GFP(+) cell population (derived from A549 or SKOV3 cells) shows a near 2-fold increase in migration, as compared with the GFP(-) cells. p<0.05 for the A549 and p<0.001 for the SKOV3 cells (Student's t-test). **B**. Effects of mitochondrial inhibitors. To assess whether mitochondrial function is involved in cancer cell migration, mitochondrial inhibitors (either oligomycin A or XCT790) were placed in both the upper and lower chambers. Note that oligomycin A and XCT790 both effectively inhibited migration in the GFP(+) cell population. Results are shown as the mean ± SEM, *t*-test, two-tailed test. *p < 0.05.

hTERT-high cells also appear to be more migratory, as observed by quantitatively comparing the migration of the GFP(+) and GFP(-) cell populations (Figure 8A). To test if this phenotype is strictly dependent on intact mitochondrial function, we examined the effects of two mitochondrial inhibitors, namely oligomycin and XCT790. Note that treatment with these mitochondrial inhibitors is sufficient to significantly reduce migration in the hTERT-high CSCs (Figure 8B, 8C).

### hTERT-high CSCs are highly proliferative and can be effectively targeted with the CDK4/6 inhibitor palbociclib

**Figure 9:**
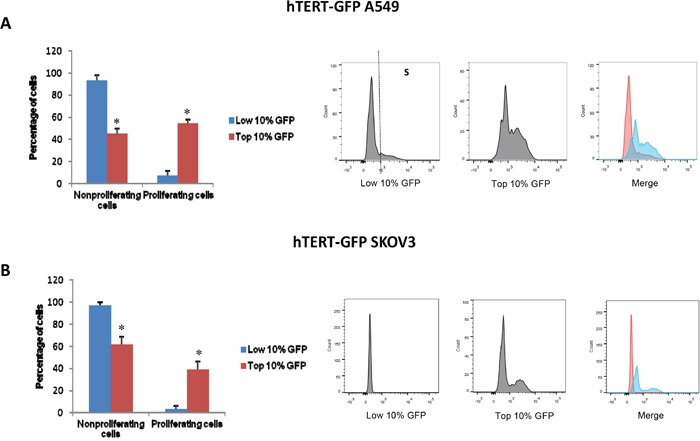
hTERT-high cells are highly proliferative and show an increased capacity for DNA-synthesis We used EdU incorporation to monitor DNA-synthesis (S-phase) in the GFP(+) and GFP(-) cell populations. More specifically, cells were treated with 10 uM EdU for 90 minutes and its incorporation was detected according to the recommended staining protocol. Briefly, for the detection of EdU, we used 405 nm excitation with a violet 450/50 nm bandpass emission filter. Measurement of total DNA content using the flow cytometer was achieved by using Pacific Blue (picolyl azide). In A549 cells, the GFP(+) population was 8-fold more proliferative than the GFP(-) cell population. Similarly, in SKOV3 cells, the GFP(+) population was 12-fold more proliferative than the GFP(-) cell population. Representative histograms show the separation of proliferating (S-phase) and non-proliferating cells. Bar graphs show the mean ± SEM of three independent experiments. Asterisks (*) denote a significant change, p<0.05.

To better understand the role of hTERT in cell propagation, we assessed DNA synthesis (S-phase) in both the GFP(+) and GFP(-) cell populations, by employing EdU-incorporation and FACS analysis. Remarkably, our results indicate that the GFP(+) cell population is the predominant proliferating cell population, whereas the GFP(-) population does not undergo significant proliferation (Figure 9). Importantly, these results directly show that the hTERT-high cells are the most significant proliferating cell population and, therefore, they should be targeted therapeutically.

**Figure 10:**
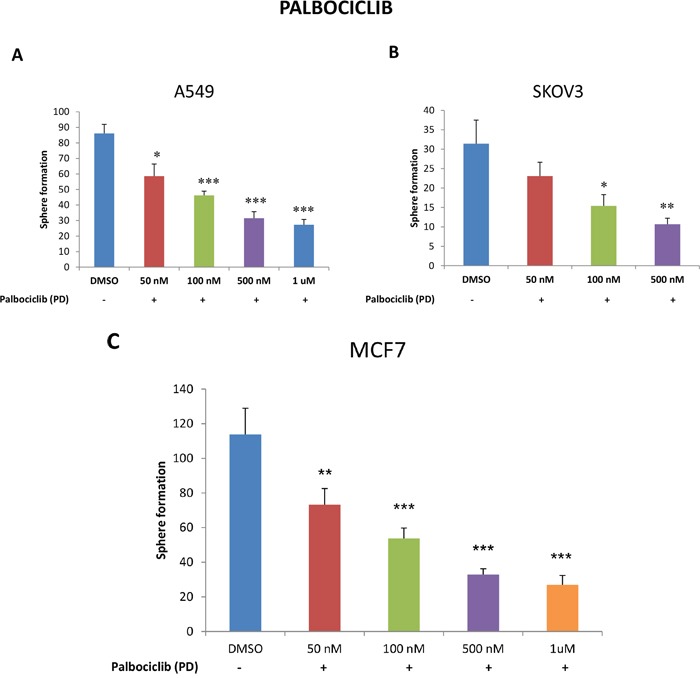
Targeting CSC propagation with palbociclib, a CDK4/6 inhibitor, in lung, ovarian and breast cancers Parental A549 and SKOV3 cells were seeded in low-attachment plates and treated with the indicated concentrations of PD-0332991 (palbociclib) for five days. Then, the number of 3D-spheroids formed was quantitated. Note that palbociclib treatment very effectively inhibited 3D-sphere formation in A549 (**panel A**) and SKOV3 cells (**panel B**), with an IC-50 < 100 nM. Virtually identical results were also obtained with MCF7 cells (**panel C**). Data represent three independent experiments. Results are shown as the mean ± SEM, *t*-test, two-tailed test. Asterisk (*) depicts significant change, p<0.05. *p < 0.05, **p < 0.005, ***p < 0.0001.

To this end, we next evaluated the effects of a well-established CDK4/6 inhibitor, namely palbociclib, on CSC propagation in A549, SKOV3 and MCF7 cells. For this purpose, we used 3D-spheroid formation as a measure of CSC-activity. Figure 10 (panels A-C) illustrates that palbociclib significantly inhibits the propagation of CSCs, in lung, ovarian and breast cancer cell lines, with an IC-50 near 100 nM. Thus, palbociclib and other CDK4/6 inhibitors may be useful to clinically target the proliferative CSC population.

## DISCUSSION

**Figure 11:**
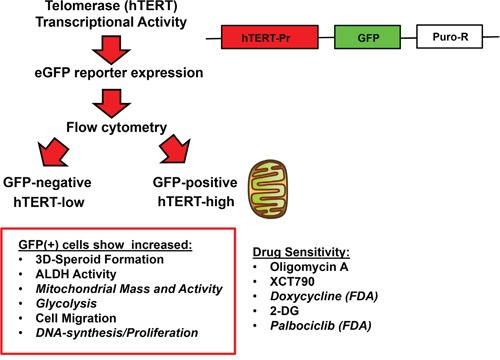
Telomerase-driven cancer stem-like cells are characterized by an anabolic, migratory and highly proliferative cellular phenotype This schematic diagram summarizes the isolation of two distinct populations of cancer cells, based on telomerase transcriptional activity. A schematic diagram of the hTERT-Promoter-eGFP-Puro-R reporter construct is shown on the right. Note that the GFP(+) cell population shows significant increases in “stemness”, metabolic activity (both oxidative and glycolytic), cell migration and proliferation. As a consequence, the propagation of this cell population can be effectively targeted with metabolic inhibitors (oligomycin A, XCT790, doxycycline, 2-DG) or by using a CDK4/6 inhibitor (palbociclib).

Here, we explored the contribution of hTERT-activity to the generation and maintenance of the CSC population, using ovarian and lung cancer cell lines as a model system. To this end, we employed a sensitive reporter system to monitor telomerase functionally, by placing the hTERT endogenous promoter sequence upstream of eGFP. Then, we isolated hTERT-high cells by FACS, by collecting the GFP(+) cell population. The GFP(-) cell population served as a critical internal control for these studies. Most remarkably, hTERT-high cells showed significant increases in both glycolytic metabolism and mitochondrial-driven oxygen consumption. This was indeed confirmed and validated further by employing proteomics analysis. hTERT-high cells also showed an enhanced ability to undergo anchorage-independent cell growth (3D-spheroid formation), as well as cell migration [13]. Both of these cellular phenotypes have been previously shown to be associated with increased “stem cell activity” in CSC populations. Importantly, these “stemness” phenotypes were effectively inhibited by treatment with compounds that halt mitochondrial metabolism (i.e., oligomycin, XCT790, doxycycline) [13–15] or ii) glycolysis (i.e., 2-deoxy-glucose) [16, 17]. Surprisingly, hTERT-high cells were highly proliferative, as evidenced by a dramatic increase in their capacity to undergo DNA-synthesis, when measured via EdU incorporation. Similarly, palbociclib (an FDA-approved CDK4/6 inhibitor) [18, 19] dramatically blocked the propagation of CSCs, as measured using 3D-spheroid formation as a functional assay, with an IC-50 in the range of 100 nM. Overall, our findings provide a new mechanistic appreciation for how telomerase activity contributes as a driver of tumor metabolic heterogeneity (Figure 11). Most notably, hTERT-high cells were the most energetic, migratory and proliferative cell population. Thus, FDA-approved drugs (such as doxycycline and palbociclib) should be considered as new therapeutic strategies for targeting hTERT-high cells, in various cancer types (Figure 11).

Similarly, several reports have now directly shown a connection between hTERT function and mitochondrial activity. This occurs through a telomerase and p53-based signaling pathway, which converges on PGC1A/B, a major mitochondrial transcription factor [20–22]. Specifically, aging-related studies directly demonstrate that impairment of hTERT function leads to mitochondrial decline, via p53 over-expression [23, 24], which ultimately inactivates PGC1A/B function [25]. Just the opposite occurs during cancer development, where p53 is often inactivated resulting in activation of PGC1A and mitochondrial function [26]. Thus, our current studies are consistent with a functional link between hTERT-high cells and mitochondrial metabolism, driving increased metabolic activation of this highly proliferative cell population.

**Figure 12:**
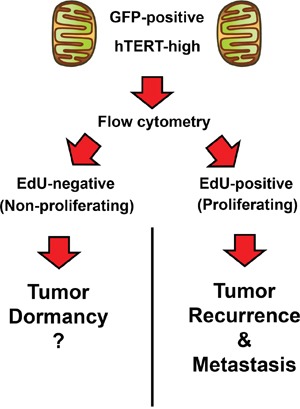
Telomerase-driven cancer stem-like cells: Are they also involved in tumor dormancy? Here, we observed that the hTERT-high cell population could be further divided into i) proliferating and ii) non-proliferating sub-populations, using EdU incorporation to monitor DNA-synthesis (S-phase). We speculate that the quiescent hTERT-high CSCs could be involved in tumor dormancy. See the Discussion section for further elaboration on this point.

Perhaps most unexpected is our observation that the CSC population is highly proliferative and can be targeted with a CDK4/6 inhibitor. However, this should not be too surprising, because telomerase activity is functionally linked to cell immortalization and proliferation [27], as well as escape from cell cycle arrest and senescence. Moreover, these experiments did allow us to distinguish two different cellular populations of hTERT-high cells, i) one that was proliferative and ii) the other that was non-proliferative (Figure 12). We speculate that the non-proliferative population of hTERT-high cells may be involved in tumor dormancy [28] and could be re-activated with the appropriate stimulus, driving tumor recurrence and metastasis [29]. Most importantly, our current work provides a novel strategy to potentially isolate the non-proliferative population of hTERT-high cells, allowing further mechanistic studies on tumor dormancy *in vitro*.

Palbociclib (PD-0332991; from Pfizer) was first approved by the FDA on February 3, 2015 as the first-line treatment for advanced post-menopausal ER+, HER2-negative breast cancer, in combination with letrozole [30]. It is an orally bioavailable and potent second generation CDK4/6 inhibitor. Mechanistically, it induces a G1-phase arrest in cell cycle progression. Remarkably, palbociclib nearly doubled progression-free survival (PFS) in this patient population (from 10.2 months to 20.2 months) [30]. Thus, palbociclib received designation as a ‘Breakthrough Therapy’ and gained accelerated approval from the FDA. There are currently at least 12 other palbociclib trials in breast cancer patients that are now ongoing, most of which are targeting advanced and metastatic disease states [30]. And, other CDK inhibitors are also being clinically developed to target metastatic disease: Ribociclib (LEE011; Novartis) and Abemaciclib (LY2835219; Eli Lilly) [30]. Since metastatic disease is thought to depend on the survival and propagation of CSCs, then it is perhaps not surprising that we observed that palbociclib can also be used to target the proliferative CSC population, in lung, ovarian and breast cancer cell lines, effectively halting 3D-spheroid formation, with an IC-50 near 100 nM. Therefore, new clinical trials using palbociclib to target the proliferative CSC population in multiple cancer types may be warranted.

Previously, we showed that palbociclib essentially behaves as a *bonafide* inhibitor of telomerase function in normal human fibroblasts [31]. More specifically, we treated hTERT-immortalized fibroblasts with palbociclib (0.1, 0.5 and 1 μM) for a period of 36 hours, and then cell lysates were prepared and subjected to immunoblot analysis. Importantly, treatment of hTERT-immortalized fibroblasts with palbociclib dramatically inhibited RB-phosphorylation, as expected, resulting in the up-regulation of markers of cell cycle arrest, such as CDK inhibitors (p16 and p21), as well as markers of senescence (β-galactosidase) and autophagy (LC3-I/II) [31]. Thus, the pharmacological induction of cell cycle arrest with palbociclib, a CDK4/6 inhibitor, is indeed sufficient to overcome the state of replicative immortality, normally conveyed by hTERT expression in fibroblasts. Similar results were also obtained by acute amino acid starvation of hTERT over-expressing fibroblasts, possibly explaining the positive benefits of caloric restriction as an anti-cancer therapy [31]. Thus, palbociclib could be functionally repurposed as an FDA-approved “telomerase inhibitor”, to target replicative immortality in cancer stem-like cells. Similarly, other groups have shown that the treatment of cancer cell lines with palbociclib induces the senescence phenotype [32, 33].

In summary, here we have used an hTERT-eGFP reporter system to study the functional role of telomerase activity in generating tumor metabolic heterogeneity. We observed that hTERT-high CSCs, are more energetically active and show an increased capacity for migration and cell proliferation. We propose several new therapeutic strategies for targeting CSCs, based on this systematic phenotypic analysis, by employing FDA-approved drugs such as doxycycline and palbociclib. These approaches mechanistically target energy metabolism and replicative immortality in CSCs.

## MATERIALS AND METHODS

### Materials

Non-small cell lung cancer (A549) and ovarian cancer (SKOV3) cell lines were obtained commercially. The hTERT-eGFP lenti-viral transcriptional reporter was custom made to our specifications by GeneCopoeia, and was as we previously described. Briefly, it contains the 1.5 kB hTERT promoter region for regulating eGFP expression and a puromycin-resistance cassette as a selectable marker for deriving stably-transduced cell populations. Similarly, ER(+) breast cancer cells (MCF7) were purchased from ATCC. Media for cell cultures (DMEM, D6546) was from Sigma-Aldrich. Cell culture media (DMEM/F12) for spheroid culture was purchased from Life Technologies. XCT790 was purchased from Tocris. The telomerase inhibitor (MST-312), oligomycin A, doxycycline and the CDK4/6 inhibitor palbociclib (PD-0332991) were all obtained from Sigma-Aldrich.

### Generation of A549 and SKOV3 lines, harboring the hTERT-GFP reporter

A549 and SKOV3 cell lines were stably-transduced with the viral supernatants obtained by transfecting packaging cells with the construct containing GFP under the transcriptional control of the hTERT promoter region (hTERT-GFP). After transduction, cancer cell lines were selected with puromycin for two weeks to generate hTERT-GFP-A549 cells and hTERT-GFP-SKOV3 cells.

### Tumor 3D-spheroid culture

A single cell suspension was prepared using enzymatic (1x Trypsin-EDTA, Sigma Aldrich, #T3924), and manual disaggregation (25 gauge needle) [34]. Briefly, 2,500 hTERT-GFP A549 cells or 5,000 hTERT-GFP SKOV3 cells were plated with spheroids medium (DMEM-F12/B27/20ng/ml EGF/PenStrep), under non-adherent conditions, in six wells plates coated with 2-hydroxyethylmethacrylate (poly-HEMA, Sigma, #P3932). Cells were grown for 5 days and maintained in a humidified incubator at 37°C with 5% (v/v) carbon dioxide/air. After five days of culture, the number of spheres >50 μm were counted.

### Flow cytometry analysis and sorting

Cells were subjected to flow cytometric analysis using the Fortessa™ X-20 and the BD Accuri™ C6. The GFP(+) (top 10%) and GFP(-) (lowest 10%) cell populations were separated and collected by using the BD FACSAria™ III and BD Influx™ cell sorter. Data were analyzed using FlowJo software (Tree Star, Ashland, OR).

### AldeRed ALDH detection assay

The AldeRed with 588-A ALDH Detection Assay (SCR150, Millipore) was used, following the manufacturer's instructions.

### Mitochondrial staining of live cells with MitoTracker probes

To evaluate the mitochondrial mass and activity, adherent cells were stained with MitoTracker Deep Red (M22426, Life Technologies) or in suspension with MitoTracker Orange CM-H2TMRos (M7511, Life Technologies), respectively. Lyophilized MitoTracker was first dissolved in DMSO to generate a 1 μM stock solution that was diluted into serum-free DMEM at a final concentration of 25 nM and 100 nM respectively. Briefly, cells were cultured for 48 hours and then stained with MitoTracker for 15 minutes at 37°C. Cells were washed in PBS and analysed by FACS.

### Immunofluorescence analysis

After six days of treatment with MST-312, the cells were subjected to immunofluorescence analysis. Briefly, the cells were incubated with MitoTracker Deep Red for 20 minutes, and, after washing, were fixed in 4% paraformaldehyde for 15 minutes. Then, cells were permeabilized for 10 minutes with PBS containing 0.2% BSA and 0.1% Tween-20. Next, a blocking step with 5% of BSA in PBS was followed by an incubation of 90 minutes with the GFP Tag Antibody, conjugated to the Alexa Fluor 488 (A21311, Life Technologies). Finally, slides were washed, stained with DAPI and mounted. Images were acquired using a fluorescent microscope.

### Seahorse metabolic flux analysis

Extracellular acidification rates (ECAR) and oxygen consumption rates (OCR) were measured using the Seahorse XFe96 bioenergetic analyzer (Seahorse Bioscience, MA, USA). GFP(+) and GFP(-) cell populations were maintained in DMEM supplemented with 10% FBS (Fetal Bovine Serum), 2 mM GlutaMAX, and 1% Pen-Strep. Fifteen thousand cells were seeded for A549 and ten thousand for SKOV3 into XF96-well cell culture plates per well, and incubated at 37°C in a 5% CO2 humidified atmosphere. After 20 hours, cells were washed in pre-warmed XF assay media, as previously described [13]. ECAR and OCR measurements were normalized by cell number. Data sets were analyzed using Seahorse XFe-96 software and Excel software, using Student t-test calculations. All experiments were performed with six replicates each, and repeated three times independently.

### Semi-quantitative proteomics analysis

Cell lysates were prepared for trypsin digestion by sequential reduction of disulphide bonds with TCEP and alkylation with MMTS. Then, the peptides were extracted and prepared for LC-MS/MS. All LC-MS/MS analyses were performed on an LTQ Orbitrap XL mass spectrometer (Thermo Scientific, San Jose, CA) coupled to an Ultimate 3000 RSLCnano system (Thermo Scientific, formerly Dionex), The Netherlands). Xcalibur raw data files acquired on the LTQ-Orbitrap XL were directly imported into Progenesis LCMS software (Waters Corp., Milford, MA, formerly Non-linear dynamics, Newcastle upon Tyne, UK) for peak detection and alignment. Data were analyzed using the Mascot search engine. Five technical replicates were analyzed for each sample (GFP(+) versus GFP(-); top 10% versus lowest 10%).

### Migration assay

Transwell-24 wells with uncoated permeable support and 8-μm pores were used (Corning). Thirty-thousand (A549) or twenty-thousand (SKOV3) viable tumor cells were seeded per well, in the upper chamber and incubated in 5% CO2 at 37°C. The upper side of the filter was filled with DMEM medium containing 0.1% BSA. The lower chamber was filled with complete culture medium containing 10% FBS as chemo-attractant. After an overnight period, non-migrated cells were removed from the upper surface of the membrane by scrubbing with cotton swabs. Inserts were stained with crystal violet (HT90132, Sigma-Aldrich). All functional experiments were performed in triplicates. Values for migration were obtained by counting five fields per membrane (objective 40X) and represent the average of three independent experiments. Oligomycin A and XCT790 were placed in both lower and upper chambers.

### Proliferation assay

The Click-iT^R^ Plus EdU Flow Cytometry Assay was used for determining the percentage of S-phase cells (DNA synthesis) in the cell populations (Pacific Blue™ picolyl azide, C10636, Molecular Probes Life Technologies), according to the manufacture's protocol.

### Statistics

Data are presented as the mean ± SEM. The student's t-test and ANOVA were used where appropriate. P < 0.05 was considered statistically significant.

## SUPPLEMENTARY TABLE




