# Supplementary material for: Targeting cancer stem cell propagation with palbociclib, a CDK4/6 inhibitor: Telomerase drives tumor cell heterogeneity
Source: Oncotarget. 2016 Dec 25;8(6):9868–84. doi: 10.18632/oncotarget.14196 (PMC5354777; doi:10.18632/oncotarget.14196)
Supplement: Supplementary file 1 [file oncotarget-08-9868-s001.pdf]

Targeting cancer stem cell propagation with palbociclib, a CDK4/6 inhibitor: Telomerase drives tumor cell heterogeneity

SUPPLEMENTARY TABLE

Supplementary Table 1: Molecules Commonly Upregulated in hTERT-GFP(+) Ovarian, Lung and Breast CSCs

See Supplementary File 1
