# Supplementary material for: Targeting cancer stem cell propagation with palbociclib, a CDK4/6 inhibitor: Telomerase drives tumor cell heterogeneity
Source: Oncotarget. 2016 Dec 25;8(6):9868–84. doi: 10.18632/oncotarget.14196 (PMC5354777; doi:10.18632/oncotarget.14196)
Supplement: Supplementary file 2 [file oncotarget-08-9868-s002.docx]

**Supplemental Table 1. Molecules Commonly Upregulated in hTERT-GFP(+) Ovarian, Lung and Breast CSCs.**

**Symbol Description Fold-Upregulation**

**(GPF(+)/GFP(-))**

**Mitochondrial-related proteins SKOV3 A549 MCF7**

**MT-CO2** Cytochrome c oxidase subunit 2, mt-DNA encoded 6.08 3.99 20.48

**PRKDC** DNA-dependent protein kinase catalytic subunit 5.90 6.26 2.25

**HSPD1** 60 kDa heat shock protein, mitochondrial 5.44 1.98 3.30

**ATP5B**  ATP synthase subunit beta 4.54 9.48 7.76

**AK2**  Adenylate kinase 2, mitochondrial 2.41 27.20 4.00

**HSPA9** Stress-70 protein, mitochondrial 2.38 6.00 N/A

**Glycolysis**

**LDHB**  L-lactate dehydrogenase B 3.08 50.93 N/A

**Glycogen-related**

**PYGB** Glycogen phosphorylase, brain form 1.63 2.23 1.59

**_______________________________________________________________________________________________________________**

**Proteomic analysis of hTERT-GFP SKOV3, A549 and MCF7 cells.** Data are derived from the analysis of FACS sorted cell populations. The fold-increase of certain key molecules in GFP(+) cells is shown, relative to GFP(-) cells. Note that several protein targets related to mitochondrial metabolism and glycolysis are increased in GFP(+) cells. For analyses of A549 and SKOV3 cells, see Tables 1 and 2. The MCF7 cell proteomics data are reproduced here from Reference 11. N/A, not applicable.
